# The potential value of serum chemerin in patients with breast cancer

**DOI:** 10.1038/s41598-021-85986-w

**Published:** 2021-03-22

**Authors:** Yanfang Song, Xianjin Zhu, Zhen Lin, Li Luo, Dan Wen

**Affiliations:** 1 Department of Clinical Laboratory, Affiliated People Hospital of Fujian University of Traditional Chinese Medicine, 602 Bayiqi Road, Fuzhou, 350001 Fujian China; 2 Department of Clinical Laboratory, Fujian Medical University Union Hospital, 29 Xinquan Road, Fuzhou, 350001 Fujian China; 3 Department of Emergency Surgery, Affiliated People Hospital of Fujian University of Traditional Chinese Medicine, 602 Bayiqi Road, Fuzhou, 350001 Fujian China

**Keywords:** Breast cancer, Cancer screening

## Abstract

Breast cancer (BC) is one of the most dangerous malignant diseases in females. However, the reliable serum biomarkers of BC still need to be explored. Chemerin levels have been found to be associated with different types of cancer. This study aimed to evaluate the role of serum chemerin as a biomarker of BC diagnosis, as well as the correlation between serum chemerin levels and clinicopathological features. The serum from 248 BC patients, 30 breast benign tumor patients, and 103 healthy controls were collected and serum chemerin levels were determined with enzyme-linked immunosorbent assay. We found that serum levels of chemerin in BC patients were higher than those in healthy control individuals (*p* < 0.05). The area under the ROC curve (AUC) for chemerin, CA15-3 and CEA was 0.703, 0.662 and 0.581, respectively, in distinguishing between breast cancer patients from healthy individuals, and the chemerin cutoff value was 100.327 ng/ml with a sensitivity of 56.60% and a specificity of 98.10%. The AUC for chemerin + CA15-3 was 0.822, which was higher than that for chemerin + CEA and CEA + CA15-3. Moreover, serum levels of chemerin were significantly associated with histologic grade, Ki67 expression, and menopausal status. However, no significant association was found between serum levels of chemerin and age, tumor size, metastase, ER status, PR status, and HER-2 status. Overall, our study suggested that the combination of chemerin with CA15-3 achieves relatively better diagnostic performance in the breast cancer. Elevated serum chemerin is associated with Ki67 expression levels and histologic grade.

## Introduction

Breast cancer is one of the most dangerous malignant diseases as well as the first major cause of cancer death in women with solid tumors worldwide^1^. In recent years, with the development of China's economy and the changes of life way, morbidity and mortality of breast cancer continue to rise in China^2,3^. At present, some molecular markers are employed for the prediction of clinical outcomes of breast cancer, including estrogen receptor (ER), progesterone receptor (PR), human epidermal growth factor receptor 2 (HER-2), and so on^4,5^. Unfortunately, at present, there is not a reliable and valuable serum biomarker for breast cancer diagnosis. Considering that serum sample from patients is easy to obtain with low invasion and serum biomarkers are simple to measure in the clinic, it is of great importance to find a reliable and valuable serum biomarker to improve the diagnosis of breast cancer.

Accumulating evidence indicated that breast tissues are mainly constituted by adipose tissue^6^. As an endocrine tissue, adipose tissue can produce a lot of adipokines^7^. A growing number of studies have shown that adipokines are related to breast cancer^2,3,7^. Chemerin, which is a novel and multifunctional adipokine, can not only mediate the chemoattraction of NK cells, macrophages, dendritic cells, but also participate in adipogenesis, immunity, angiogenesis, and metabolic activity through binding to G-protein-coupled receptors^8,9^.

Recently, more and more studies focus on the relationship between chemerin and cancers and found that chemerin acts an essential role in cancers^10^. Chemerin expression is different depending on the tumor type^10^. Chemerin has been shown to be downregulated compared to normal tissue counterparts in hepatocellular carcinoma^11^, melanoma^12^, non-small cell lung cancer^13^, adrenocortical carcinoma^14^, prostate cancer^15^, and acute myeloid leukemia^16^. In contrast, chemerin is significantly upregulated in glioblastoma^17^, mesothelioma, and squamous cell carcinoma^18^. Interestingly, some studies found that serum/plasma chemerin levels are related to clinical outcomes in hepatocellular carcinoma^19^, adrenocortical carcinoma^14^, gastric cancer^20^, and non-small cell lung cancer^13,21^. These findings suggest that serum/plasma chemerin is a promising prognostic biomarker for certain tumors.

To our knowledge, the clinical significance of chemerin in breast cancer has been only reported by a very limited number of studies. In 2018, El-Sagheer et al. found that chemerin expression is increased in breast cancer tissues, and high chemerin expression is associated with poor prognosis and unfavorable clinical and pathological parameters^22^. Thus, serum chemerin levels may be a promising prognostic biomarker in breast cancer. A recent report has shown that serum chemerin levels are not correlated with breast cancer stage^23^, but the results of this report about the role of serum chemerin in breast cancer are limited because the sample size is small and no healthy controls are found^23^. Therefore, the clinical significance of serum chemerin in breast cancer is needed to further research with larger sample sizes.

Therefore, in the present study, serum chemerin levels were detected and the potential value of serum chemerin as a biomarker was explored in breast cancer patients. The results showed that serum chemerin levels were elevated in breast cancer patients. Moreover, we observed that serum chemerin has a better diagnostic performance than serum CEA and serum CA15-3 in breast cancer. Importantly, serum levels of chemerin were significantly associated with Ki67 expression and histologic grade of breast cancer patients. Our findings indicated that combination chemerin and CA15-3 is a promising candidate serum biomarker in breast cancer.

## Results

### Serum levels of chemerin, CEA, and CA15-3 elevated in patients with breast cancer

**Figure 1:**
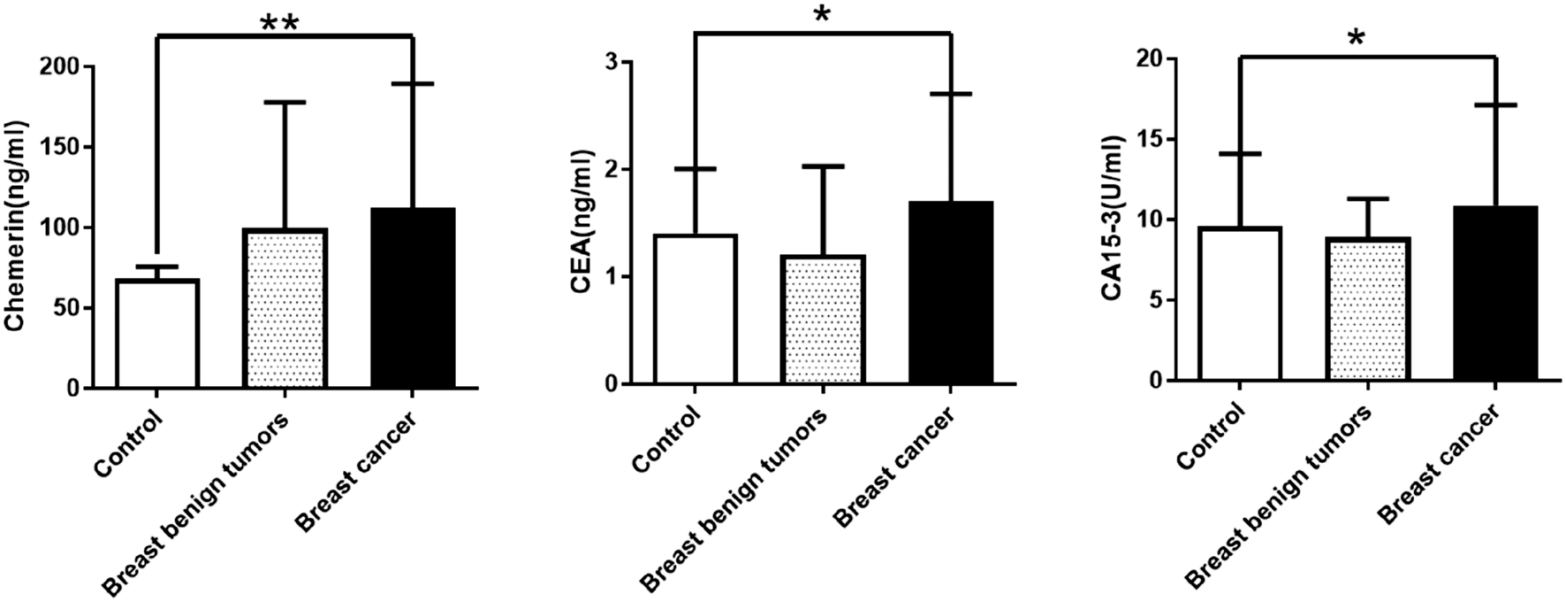
Serum levels of chemerin, CEA, and CA15-3 in breast cancer patients, breast benign tumors, and healthy controls. The data are presented as median with IQR. **p < 0.01, *p < 0.05.

Serum levels of chemerin in 248 patients with breast cancer patients, 30 patients with breast benign tumors, and 103 healthy controls were determined. The results showed that serum levels of chemerin, CEA, and CA15-3 were significantly higher in breast cancer patients than in healthy controls. However, no significant difference was found in serum levels of chemerin in breast benign tumors and healthy controls (Fig. 1). Therefore, our data showed that serum levels of chemerin, CEA, and CA15-3 are significantly elevated in breast cancer patients.

### Evaluation of the diagnostic power of serum chemerin in breast cancer

**Figure 2:**
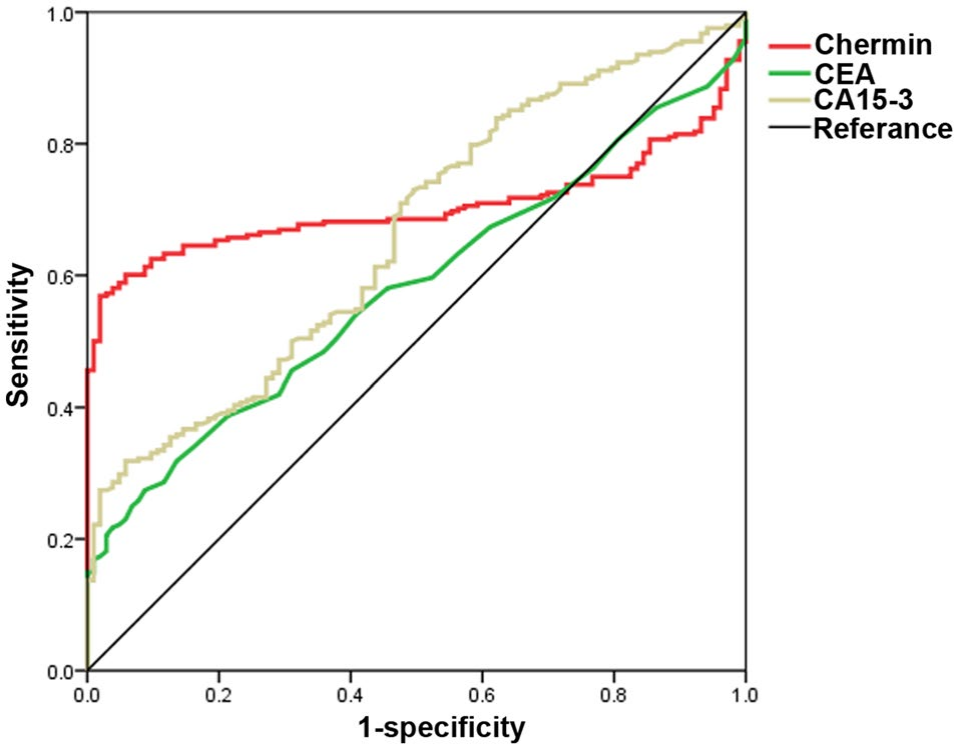
Receiver operating characteristic curves of chemerin, CEA, and CA15-3 in distinguishing breast cancer patients from healthy controls.

ROC curve analysis was used and the results showed that the area under the ROC curve (AUC) for chemerin, CEA and CA15-3 was 0.703, 0.581 and 0.662, respectively, in distinguishing between breast cancer patients from healthy individuals, and the chemerin cutoff value was 100.327 ng/ml with a sensitivity of 56.60% and a specificity of 98.10% (Fig. 2 and Table 1). Moreover, we found that the AUC for chemerin + CA15-3 was 0.822, which was higher than that for chemerin + CEA and CEA + CA15-3 (Table 1). These outcomes indicated that chemerin could enhance the diagnostic power of CA15-3 and CEA, and combination chemerin and CA15-3 has better diagnostic performance in the breast cancer patients.

**Table 1:** Diagnostic value of Chemerin, CEA and CA15-3 for breast cancer patients.

Variables	AUC	Cut off	Sensitivity (%)	Specificity (%)	95% confidence interval	
					Upper limit	Lower limit
Chemerin	0.703	100.327	56.60	98.10	0.767	0.642
CEA	0.581	2.45	27.40	91.30	0.641	0.521
CA15-3	0.662	16.97	31.90	94.20	0.721	0.602
Chemerin + CEA	0.768		59.70	97.10	0.815	0.720
Chemerin + CA15-3	0.822		65.30	97.10	0.864	0.780
CEA + CA15-3	0.691		39.90	92.20	0.748	0.634
Chemerin + CEA + CA15-3	0.838		66.50	98.10	0.878	0.798

### Correlation between serum levels of chemerin and clinicopathological features in breast cancer patients

On the basis of chemerin cut-off value, patients were divided into chemerin-high (> 100.337 ng/ml) and chemerin-low groups (≤ 100.337 ng/ml). Because the clinicopathological characteristics of some patients is missing, we analyze the relationships between chemerin levels and clinicopathological characteristics of 177 BC patients. The results showed that serum levels of chemerin were significantly associated with histologic grade, Ki67 expression levels, and menopausal status (Table 2), and serum levels of chemerin in breast cancer patients with ER-negative, PR-negative, or HER-2-negative were higher than those with ER-positive, PR-positive, or HER-2-positive, respectively, but these results were not statistically significant. No significant association was shown between serum chemerin levels and age, body mass index (BMI), tumor size, and metastases (Table 2).

**Table 2:** Relationship between Chemerin and pathological characteristics in breast cancer patients.

Variables	Total	Chemerin ≤ 100.337 ng/ml	Chemerin > 100.337 ng/ml	*p*
		n (%)	n (%)	
Sample size	177	78 (44.0)	99 (56.0)	
**Age**				0.509
≤ 44	50	24 (48.0)	26 (52.0)	
> 45	127	54 (42.5)	73 (57.5)	
**BMI(kg/m**^**2**^)*				0.593
< 25	132	61 (46.2)	71 (53.8)	
≥ 25	41	17 (41.5)	24 (58.5)	
**Tumor size (cm)**				0.718
≤ 2	79	36 (45.6)	43 (54.4)	
> 2	98	42 (42.9)	56 (57.1)	
**Metastatic status**				0.493
No	97	45 (46.4)	52 (53.6)	
Yes	80	33 (41.4)	47 (58.6)	
**Histologic grade**				**0.012**
I	14	7 (50.0)	7 (50.0)	
II	105	54 (51.4)	51 (48.6)	
III	58	17 (29.3)	41 (70.7)	
ER status*				0.720
Negative	38	16 (42.1)	22 (57.9)	
Positive	90	41 (45.6)	49 (54.4)	
PR status*				0.435
Negative	52	21 (40.4)	31 (59.6)	
Positive	76	36 (47.4)	40 (52.6)	
HER-2 status*				0.214
Negative	41	15 (36.6)	26 (63.4)	
Positive	87	42 (48.3)	45 (51.7)	
Ki67 levels*				**0.045**
≤ 20	66	34 (53.7)	29 (46.3)	
> 20	63	24 (32.4)	42 (63.6)	
**Menopausal status**				**0.020**
Premenopausal	104	56 (55.8)	48 (46.2)	
Postmenopausal	73	22 (38.6)	51 (61.4)	

*Data of some patients is missing. Bold indicates a statistically significant.

## Discussion

To the best of our knowledge, this is the first report that has addressed serum levels of chemerin from breast cancer patients and healthy individuals, and evaluated its role for the diagnosis and prognosis of patient with breast cancer. In the present study, we found that serum levels of chemerin in breast cancer patients were increased. ROC analysis showed that combination chemerin with CA15-3 had better diagnostic power than serum CEA alone and serum CA15-3 alone in breast cancer diagnosis. Moreover, serum levels of chemerin were significantly associated with histologic grade and Ki67 expression of breast cancer patients. These results indicated that chemerin has a potential role as a serum biomarker in breast cancer.

As a novel adipokine, chemerin is known to function in mediating angiogenesis, cell proliferation, and migration, immunity through binding to G-protein-coupled receptors^8,9^. Recently, increasing studies found that chemerin has a significant role in the diagnosis, prognosis, and development of cancer^7,24^. In 2018, El-Sagheer et al. found that chemerin expression in breast cancer tissue is higher than in the corresponding normal breast tissue^22^. However, there is only a study about the relationship between serum chemerin and breast cancer, but the authors do not compare serum chemerin levels in breast cancer with healthy controls due to lacking of healthy controls^23^. In the present study, we firstly found that, compared with healthy controls, serum chemerin levels were increased in breast cancer patients. As we know, serum from patients is easy to collect and serum biomarkers are simple to measure. Unfortunately, to this day, there is not a reliable serum biomarker for the diagnosis of breast cancer. In our research, the combined detection of chemerin with CA15-3 has better diagnostic performance in discriminating BC patients from healthy participants, with higher sensitivity and specificity. To our knowledge, our study is the first to report the clinical utility of the combination of chemerin and CA15-3 for the diagnosis of patients with BC.

A number of clinical studies have shown that breast cancer patients with high histologic grade, Ki67 expression levels, or metastasis are thought to have a poor prognosis^4,5^. We analyzed the relationships between serum chemerin levels and clinicopathological characteristics of breast cancer, and the results showed that serum levels of chemerin were associated with histologic grade, these findings are consistent with El-Sagheer’s report, in which chemerin expression in breast cancer tissues is significantly correlated with tumor grading^22^. Ki-67, which is a nuclear protein, reflects the cell proliferation of breast cancer^24^. In the present study, we found that serum levels of chemerin in breast cancer patients were associated with Ki67 expression in breast cancer tissues. The mechanism that could explain these findings is unclear. We speculated that the mechanism may be related to the angiogenic functions of chemerin. Earlier reports have revealed that chemerin can promote angiogenesis by activating the production and activity of matrix metalloproteinases, and angiogenesis contributes to the proliferation of breast cancer cells^20,24,25^. Considering the key role of Ki67 expression levels and high histologic grade in predicting clinical outcome of breast cancer^4,5^, our finding suggests that, in the absence of tissue samples, serum chemerin, as an inexpensive and easily obtainable parameter, may play an auxiliary role in the prognostic evaluation of BC.

Some shortcomings of this study should be acknowledged. First, the sample size in this study is relatively small, which might raise the bias of analysis. Second, this study does not determine the accuracy of chemerin's diagnostic performance in patients with suspected breast cancer. Third, no data on overall survival and the effectiveness of treatments was obtained, thus we don’t evaluate the relationship of serum levels of chemerin with overall survival and the effectiveness of treatments in breast cancer patients. In addition, we only measured the serum levels of chemerin from newly diagnosed patients in the current study, and measuring serial changes of serum chemerin in different time points before and after treatment may supply clinicians with more information. Hence, further research with large sample size studies and measuring serial changes of serum chemerin levels in different time points before and after treatment should be conducted to confirm the clinical usefulness of chemerin in breast cancer.

In summary, our study has shown for the first time that serum chemerin levels are increased in breast cancer patients. Furthermore, we demonstrated that the combined detection of chemerin with CA15-3 has better diagnostic performance in discriminating BC patients from healthy participants, and elevated serum chemerin is associated with ki67 expression and histologic grade of breast cancer. Considering that serum from patients is convenient to collect and the method of serum chemerin is easy to operate, serum chemerin could be served as a promising biomarker in breast cancer. Whether chemerin can help to improve the diagnosis and prognostic evaluation of breast cancer needs further investigation.

## Subjects and methods

### Study subjects

In this study, 248 newly diagnosed breast cancer patients (age range: 18–81 years) and 30 breast benign tumor patients (fibroadenomas, lipomas, and intraductal papillomas, age range: 18–59 years) were recruited at Fujian Medical University Union Hospital (Fuzhou, China) from January 2019 to December 2019. This study had excluded the patients who had prior history of any type of cancer. 103 healthy controls (age range: 22–78 years) are from the health examination center of Fujian Medical University Union Hospital and receive adequate screening and excluding for breast or other types of malignancies, and other disease. The clinicopathological characteristics and laboratory data of the 177 BC patients were obtained by screening the hospital medical records system. This research was approved by the ethics committee of the Fujian Medical University Union Hospital (IRB number 2019KJT094). Informed consent was obtained from all participants included in the study. All methods were carried out in accordance with relevant guidelines and regulations.

### Determination of serum chemerin levels

Blood samples were collected and centrifuged at 1500 g 10 min. Serum samples were stored at − 70 °C until the day of the analysis. Serum levels of chemerin were determined using an enzyme-linked immunosorbent assay kit (ELISA) (R&D Systems, Inc., Minneapolis, MN, USA), following the manufacturers’ protocol. Serum levels of chemerin were calculated from a standard curve based on reference standards.

### Statistical analysis

In the present study, statistical analyses were performed using SPSS software (version 21.0, SPSS Inc., Chicago, IL, USA, https://www.ibm.com/analytics/spss-statistics-software). Chemerin values are non-normal distribution and expressed as median with interquartile range (IQR). The Mann–Whitney U test was applied to analyze the difference of serum chemerin in groups. Receiver-operating characteristic (ROC) curves were used to evaluate the diagnostic value of chemerin. The chemerin cut-off value was calculated using the Youden index. The relationship between serum levels of chemerin and clinicopathological features was analyzed using chi-square test. All tests were 2-tailed and statistical significance was set at *p* value less than 0.05.
